# A Lifespan Developmental-Stage Approach to Tobacco and Other Drug Abuse Prevention

**DOI:** 10.1155/2013/745783

**Published:** 2013-04-18

**Authors:** Steve Sussman

**Affiliations:** Departments of Preventive Medicine and Psychology, Institute for Health Promotion and Disease Prevention Research, University of Southern California, Los Angeles, CA 90033, USA

## Abstract

At least by informal design, tobacco and other drug abuse prevention programs are tailored to human developmental stage. However, few papers have been written to examine how programming has been formulated as a function of developmental stage throughout the lifespan. In this paper, I briefly define lifespan development, how it pertains to etiology of tobacco and other drug use, and how prevention programming might be constructed by five developmental stages: (a) young child, (b) older child, (c) young teen, (d) older teen, and (e) adult (emerging, young-to-middle and older adult substages). A search of the literature on tobacco and other drug abuse prevention by developmental stage was conducted, and multiple examples of programs are provided for each stage. A total of 34 programs are described as examples of each stage (five-young children, 12-older children, eight-young teens, four-older teens, and five-adults). Implications for future program development research are stated. In particular, I suggest that programming continue to be developed for all stages in the lifespan, as opposed to focusing on a single stage and that developmentally appropriate features continues to be pursued to maximize program impact.

## 1. Introduction

Lifespan development refers to the neurobiological (e.g., maturation, aging), cognitive (e.g., motivation, reasoning), microsocial (e.g., family, peer group), and macrosocial-level (e.g., socio-cultural, mass media) events, and their inter-relationships, that occur across one's life. The vicissitudes in which one interacts with the world, impacting on the world and being impacted by it, is a function of one's developmental age or stage [1, 2]. Influences on individuals vary over the lifespan and may direct one's developmental course. For example, self-esteem tends to increase from adolescence until 50 years of age and then begins to decrease, and it impacts depression symptoms, relationships, job satisfaction, and to a lesser extent, physical health, throughout the life course [3]. Also, behavior-response contingencies are learned throughout the life-span, which vary as a function of environmental demands and physical capacity, and contingency-based goal striving is associated with higher self-esteem [2].

Various general theoretical stages of development have been delineated among children and adults [4, 5]. As an example, Piaget [6] observed general stages of cognitive and intellectual development that occurred during the life course of children. These include a Sensorimotor stage (development of motor coordination and object permanence, 0-1 years old), Preoperational stage (development of representational capacities, 2–5 years old), Concrete operational stage (development of an understanding of logical principles as applied to concrete and specific objects and ability to take role of others, 6–11 years old), and a Formal operational stage (development of the ability to generalize, think abstractly, and test hypotheses, 12 years old on). Relatively abstract learning in prevention programming might only be appropriate for older youth [7].

Similarly, Kohlberg [8] studied the development of moral reasoning patterns, including a Preconventional stage (during which emphasis is placed on getting rewards and avoiding punishments; applies more to young children), Conventional stage (during which emphasis is placed on social rules), and a Postconventional or Moral stage (during which there is an emphasis on moral principles and conscience; applies more to older children and teens). One may envision that prevention programming, if any, which pertains to moral principles, would only be applicable to older youth. Other theories attempt to encompass the full lifespan, including Erikson's Stages of Psychosocial Development (infancy: 1 to 18 months, basic trust; early childhood: 18 months to 3 years old, autonomy of self; play age: 3 to 5 years, initiative; school age: 6 to 12 years, industriousness; adolescence: 12 to 18 years, identity; young adulthood: 18–35 years, intimacy and solidarity; middle adulthood: 35 to 65 years, generativity; late adulthood, 65-death, integrity [9]). Thus, for example, some researchers have applied Erikson's ideas and suggested that alcohol prevention is best implemented during adolescence to help them resolve role confusion and achieve a positive identity [10].

Yet other theories focus on the adult years, such as Levinson's Life Structure Theory (early adult transition: 17 to 22 years, preliminary choices for adult-like lifestyle; entering adult world: 22–28 years, adult lifestyle in work and love; age 30 transition: 28–33, life structure shift; settling down: 33 to 40 years, establish niche; mid-life transition: 40 to 45 years, life structure questioning; entering middle to late adulthood: 45 to 50 years, commitment to later years [11]). Some researchers suggest that early adult transition, or emerging adulthood, may be a critical time for drug abuse prevention efforts [12–14], as one explores different options for an enduring adult lifestyle.

Deviations within these different stages of development delineate time points in which difficulties in functioning may occur, if not protected by preventive efforts. A nonoptimal temperament, difficulties with self-regulation, relatively low self-esteem, dysfunctional beliefs or other deflections of cognitive development, difficulties with meeting life demands, relative lack of social connectedness, or difficulty reading social cues, experienced within the context of a harsh environment, paired with availability of sources of immediate relief, may direct one away from prosocial goal striving and lead to even lower self-esteem and self-destructive behaviors including tobacco and other drug use [4, 15–18]. Many of these risk factors may be initiated during the first five years of life [4].

As with other facets of development, the etiology of tobacco or other drug misuse varies with developmental stage. One rarely observes a young child using tobacco or other drugs, although there are exceptions (e.g., young inhalant or cigarette smokers who sometimes use to decrease appetite, particularly where there is a scarcity of food). However, young children may be regularly exposed to passive smoking [19]. Risk factor variables mentioned above, such as difficulties with self-regulation or relatively low self-esteem, tend to differentiate those youngsters at risk for later tobacco or other drug use, after being exposed to others' use behavior or other risky behaviors earlier in life [18].

Risk factors and mediating processes that lead to tobacco or other drug use change over the life span [15]. Jamner and colleagues [20] suggested three developmental stages relevant to tobacco use: (a) elementary to junior high school (increasing interest in peer acceptance, wider access, and exposure to cigarettes; onset of puberty), (b) junior to senior high school (new brain connections occurring), and (c) high school to independent living. The youth who are relatively precocious may begin tobacco use during elementary school. The transition into junior high school and greater need for peer acceptance, along with social images suggesting that tobacco use might increase peer acceptance, or simply due to mere curiosity and increased access, may lead to tobacco initiation at that transition period [21]. Entry into senior high school may also be a second period in which youth may be at risk for beginning or escalating tobacco or other drug use, for social benefits [22]. Also, risky, pleasurable behaviors are likely to be most rewarding during that time when new brain connections are occurring (greater limbic system response and less neocortical inhibition [23]). When entering into adulthood, some persons may decide to terminate tobacco or other drug use, whereas others may decide that tobacco or other drug use is an important aspect of their identity, leading to continued experimental or regular use [13].

Several researchers have recognized the importance of human development when creating tobacco or other drug use prevention programming. They note that prevention programming needs to be developmentally appropriate and target behavioral irregularities at specific developmental stages that might be indicative of future substance use [15, 23]; different types of programming are relevant for youth in different age groups. That is, optimal tobacco and other drug abuse prevention programming may be differentiated as a function of age or developmental level [15, 19]. Obviously, the youth in different age groups demonstrate different reading levels, perceptual-motor development, and level of abstraction [5]. Just as school topics, media foci, parenting practices, and friendship patterns vary across age groups, so would the contents and delivery of tobacco and other drug education-type programming.

At minimum, programming needs to be tailored to the reading or functional level of the group it is provided to. Also important though, careful considerations in program design are necessary to tap key developmental-stage issues. For example, while peer use and perceived peer use play a significant role in adolescent tobacco and other substance use, this does not seem to be a relatively important predictor for preadolescent children's use compared to family use and perceived family use [24]. Also, young children who are without enduring supportive adults in their lives are at an increased risk of “acting out” and blaming others when they find themselves in conflict situations [23, 25, 26]. Family-based programming may be most relevant for younger persons, whereas instruction in assertiveness skill pertaining to peer group interactions and decision making (which involves abstract reasoning and independent thought) may be more fruitfully provided to older youths [24, 27].

Most importantly, programming needs to be appropriately timed to occur in the lifespan so as to exert a maximal impact [28]. That is, programs should target precursor behaviors occurring prior to the target outcome behavior. In this paper, I describe effective tobacco or other drug use prevention programming at different developmental stages that target precursor behaviors and the outcome behavior. I delineate the developmental stages as: young children (approximately 0–5 years old), older children (6–11 years old), young adolescents (12–15 years old), older adolescents (16-17 years old), and adults (three substages being emerging adulthood, 18–25 years old; young-to-middle adulthood, 26–50 years old; and older adulthood, 51 years old and older). The age division points certainly are not sharp boundaries and can overlap to some extent. That is, as an example, given programs may be appropriate for someone four to seven years old, 11 to 13 years old, 18 to 22 years old, or 50 to 65 years old. However, I describe general developmental changes that can be fruitfully delineated by these five stages which, in turn, suggest differences in program contents.

## 2. Materials and Methods: Literature Search

I engaged in an electronic search of the literature, using four search engines: Google Scholar, PsycINFO, OvidSP (1946 to Week 3 of November 2012), and PubMed Central. I engaged in six searches for each search engine pairing “tobacco use prevention,” “smoking prevention,” and “drug abuse prevention” with “developmental age” and “developmental stage.” While the total number of pages found (taken across search terms) were 939, 3, 0, and 46, on the four search engines, respectively, many of the pages referred to stage of tobacco or other drug use (e.g., ever tried, experimenter, regular user) as opposed to stage of human development. Only 20, 3, 0, and 7 pages (total = 30) were directly relevant to this topic. In addition, I visited the NREPP website. NREPP (National Registry of Evidence-Based Programs and Practices) is a searchable online registry of more than 250 interventions related to mental health and substance abuse prevention and treatment [29]. This registry permits searching under “substance abuse prevention” and four age groups (0–5 (early childhood), 6–12 (childhood), 13–17 (adolescent), and 18–25 (young adult)). I also examined two additional sources: Colorado Blueprints [30] and the National Institute on Drug Abuse (NIDA) Redbook-second edition [31]. A description of developmental issues and examples of tobacco and other drug use prevention programming as a function of developmental stage follows [14, 23].

## 3. Results and Discussion

### 3.1. Young Children (Approximately 0–5 Years Old)

Young children who appear hypersensitive about fulfillment of immediate needs (e.g., food and comfort) or who are not well grounded in ongoing supportive and educative interactions with significant other adults are relatively likely to resort to “acting out” as a means to express their dissatisfaction [25, 26, 32]. Manifestations of such negative conduct include impulsivity, exhibition of fluctuating affect, impatience, defiance, and negativity (e.g., tendency to blame others, diffuse hostility [16, 26, 32]. Poor family management practices and early school failure (preschool or kindergarten), conflicted social attachments and errors in reading social cues, also may be evident [4, 32]. Hawkins et al. [33] asserted that problematic conduct early in life was predictive of later problem behaviors including tobacco and other drug use. Relatively severe and frequent antisocial conduct early in life may predict earlier onset of tobacco or other drug use. Those who initiate substance use at younger ages are more likely to become dependent on drugs and incur disruption of normal processes of neurobiological development [23].

Conversely, environmental protective influences to assist youth in first bonding with family, then bonding with school authorities (e.g., teachers), and then bonding with peers will tend to deter tobacco use or other drug onset or experimentation. Thus, some researchers argue that prevention should focus on the very young, whereby those at high risk for tobacco and other drug misuse can be recognized at a young age and receive assistance while they are rapidly developing and drug use is still a distal event [34, 35]. Researchers have found early intervention beneficial in directing individuals away from problem behaviors and toward prosocial activities [36].

Prevention programming to be well matched for young children should address developmental limitations and correct for behavioral irregularities that might signify a long pathway to tobacco and other drug use. Parental education that promotes consistent, firm, and kind parenting may help to normalize child behavior [32]. Also, teaching the child self-control and helping the child to perceive a closeness with parent or guardian and early grade school teachers through meetings that provide appropriate feedback (teach conversation skills, decision making through use of dolls; proper use of timeout) may help in normalizing behavior [32]. During this period in life, children can learn general concepts pertaining to being healthy; however, application of health concepts across situations, which demands some abstraction and generalization-type cognitive skills, may be unlikely [37]. In addition, perhaps some work on resource acquisition and early academic preparation skills may assist. Programming may be limited or need alteration when applied to children or parents with special needs (traumatized, intellectually challenged). Certainly, parents should be encouraged not to smoke around their children and should, themselves, quit smoking [19]. This same admonition might be suggested regarding alcohol or other substances.

Thus, key material of instruction that might prevent future tobacco or other drug misuse in young children includes (a) emotional learning, (b) enhancement of bonding to parents and teachers, (c) self-control, and (d) possibly simple information on bad substances. I present five programs; four of them were on the NREPP website (Fast Track was not on the site [38]) (a total of eight programs were located on the NREPP website for young children). None of the programs for young children have empirically demonstrated an impact on tobacco or other drug use years later; evaluations focus on precursors of later tobacco or other drug misuse.

Regarding emotional learning, it is important to develop the ability to competently express oneself emotionally and regulate one's emotions. The *I Can Problem Solve (ICPS)* program was designed for nursery school and kindergarten age students [39]. It has been found to lead to less impulsive behavior, better problem-solving skills, and better classroom behavior in kindergarten. This 6-to-8-week classroom-based program is taught in three sections: (1) the first section focuses on using games to learn problem-solving vocabulary; (2) the second section focuses on having the children learn how to listen and to identify their feelings and those of others; and (3) the third section presents the children with hypothetical problems and they are asked to analyze these problems in regard to the feelings of those involved, examine consequences, and problem solve. Parents are also involved and taught to think about their feelings, their children's feelings, and how to help their child engage in effective problem solving. A quasi-experimental controlled trial with teacher (and parent) facilitators of low income preschool and kindergarten children has shown effectiveness for behavioral adjustment ratings, impulsivity, and self-control [40, 41] at 6-and-12-month followups, compared to no-treatment controls. When implemented among first graders in another randomized controlled trial (combined with a version of the Strengthening Families Program (SFP) for young children (the SFP “young teens” program version is described below)), ICPS showed effects compared to no treatment controls nine months later on five mediators (precursors) of substance use: school bonding, parenting skills, social competence, family relationships, and behavioral self-regulation [42].

Improving parenting is a major means of tobacco and other use prevention for young children. Activities may include linking families with health and human services, instruction in good decision making, and instruction of communication skills of mother with child and professionals. One major example of effective programming here is the *Nurse-Family Partnership (NFP)* program for 0–5-year olds [43]. This program involves registered professional nurse visits to the home of expectant mothers up to the first two years of a child's life and involves prenatal care (e.g., diet, smoking cessation), instructing good parental care of the young child, and parent education and vocation assistance. Program effects seven years after implementation of a randomized controlled trial of NFP (age 9) primarily with low income African American mothers relative to standard care (screening and transportation services) included using fewer substances (i.e., mothers' use of alcohol, marijuana, and cocaine; marginal effect), fewer documented childhood injuries, greater school readiness, and better grades at school for their children [44].

The *Fast Track Prevention Trial for Conduct Problems* runs from preschool through sixth grade but is most intensely provided during kindergarten, for children showing problem behaviors [45]. Children receive social skills training, academic tutoring, and a classroom intervention designed to enhance emotional awareness, self-control and problem solving skills, as well as activities to do at home with parents [46]. Parents are trained when their child is in the first grade on ways to foster child academic achievement (and improve communication with schools) and improve child discipline strategies (child anger control). Biweekly home visits supplement initial parent training. One randomized controlled trial which involved group and individual sessions beginning in first grade and spanning 10 years found, among a baseline high risk kindergarten group (disruptive and aggressive behavior), positive impacts on parenting behaviors, and improved peer relations, social-cognitive skills (e.g., emotion coping), and academic achievement [47]. Among the highest risk youth, intervention students demonstrated lower lifetime prevalence of conduct disorder than the control group participants, suggesting prevention more than 50% of conduct disorder cases [47]. Long-term follow-up evaluations are needed to ascertain whether or not the program indeed reduces tobacco and other substance use during childhood and adolescence. Also, it is important to see whether or not a more streamlined version of the program will show an impact as the trial was time intensive.

The *Promoting Alternative Thinking Strategies (PATHS)* classroom program was designed to reduce aggression and behavior problems (low self-control) in preschool and elementary school children [48]. The program is designed to be implemented by classroom teachers two to three times per week (30 minutes each) for approximately one year. The program is based on the “ABCD” (affective/behavioral/cognitive/dynamic) model. More specifically, child training is provided in self-control, emotional awareness, and interpersonal skills, leading to relatively improved emotional awareness, reduced anger attributions, and fewer externalizing and internalizing difficulties (this program has been used in conjunction with Fast Track [47]). For example, one study of regular second and third grade students (7–9 year olds) found greater inhibitory control and verbal fluency at one-year followup among PATHS students in a randomized trial (but of only four schools) compared to students in standard care comparison classrooms [49]. Similar types of effects have been found among the preschool and early elementary school youth, though effect sizes tend to be small for both age groups. Sessions are delivered at school three times a week for six months, so this program is time-intensive.

While this is debatable, some program developers have investigated the impact of providing simply stated information on the physical consequences of tobacco and other drug use. For example, the *Healthy Alternatives for Little Ones* program was developed for 3–6-year olds [50] and involves such activities as healthy versus harmful recognition cards. However, evidence for the efficacy of this program was limited to posttest knowledge scores compared to a wait-list control group in a quasi-experimental design, and no peer reviewed papers were produced [51].

Other programs located on the NREPP website for this age group included: Al's Pals: Kids Making Healthy Choices (instruction of and impact on self-control skills, problem solving); DARE to be You (instruction of and impact on parental competence and satisfaction, child development); Families and Schools Together (FAST; parental outreach and multifamily groups, results only reported for elementary school children on externalizing behavior and academic achievement); and Parenting Wisely (computer-based parent communication and discipline training, leading to fewer child behavior problems).

### 3.2. Older Children (Approximately 6–11 Years Old)

Older children are more influenced by family than friends (until early adolescence), though peer social influence may operate for some youth as young as nine years of age or younger [52], particularly exerted by older schoolmates [53]. Elementary school-age youth may have only just recently heard about tobacco, alcohol, and some drugs such as marijuana but probably exhibit little curiosity to try them at the present time.

The same types of strategies used for young children may apply to children 6–11 years old, provided in a more verbal-based manner (i.e., social, self-control, child academic skills training). With this age group, some facts about short-term and long-term consequences of drug use should be provided. Program contents including attribute-based similarity rather than more abstract material is likely to be relatively effective for this age group (e.g., drawing similarity between smelly socks and cigarette butts versus instruction in behavior-disease processes [7]). A greater emphasis on having the child meet with adults at institutions other than the home may be important at this age (e.g., teachers, counselors, and church leaders). Parents may need to focus on who the child's friends are and take an active role in friendship selection and monitoring. Parents should be good role models, as well as agents of socialization, for this age group (as well as other age groups). In a review of 27 evaluation studies that examined drug use prevention effects of programs designed for this age range, 56% found significant decreases in drug use [54], suggesting that programming for older children can be efficacious.

Key material of instruction that might prevent future tobacco and other drug misuse for this age group includes (a) provision of tobacco or other drugs consequences information, (b) behavioral management (to assist with youth emotional and social skills development), and (c) improvement of parenting. A total of 57 programs were on the NREPP site for this age group. Except for DARE to be You, HALO, and the Nurse-Family Partnership, the other five “young children” programs on the NREPP site overlapped with the “older children” programs. I describe 12 additional programs for older children, seven of which are on the current NREPP site (the ones that are not included are Know Your Body, Classroom-Centered (CC)/Family-School Partnership (FSP), Seattle Social Development Project, Linking the Interests of Families and Teachers, and Smoke-Free Kids.)

*BrainTrain4Kids* for 7–9 year olds is a program that examined effects and negative consequences of ATOD on the brain and body through a website [55]. The program utilizes a “scientific inquiry” method. Effects of the program on drug knowledge and drug-related attitudes have been found at immediate or 1-week delayed posttests in a randomized design compared to a wait-list control; behavior was never assessed [56].

The *Protecting You, Protecting Me (PY/PM)* program is designed for children ages 6 to 11 (Grades 1 to 5). This program focuses on the importance of protecting the brains of individuals under the age of 21 from the biological effects of alcohol as well as helping children avoid the risks associated with being in a car with inebriated drivers [57]. The program is comprised of a series of 40 lessons from grades 1 to 5 (8 lessons each grade) covering a variety of life skills including media awareness, communication, and vehicle safety. Lessons are taught by peer educators who are trained for two and a half days. One randomized design evaluation among third, fourth, and fifth graders as instructed by high school students found multiple effects in a variety of domains such as vehicle safety skills, intentions to ride with an alcohol impaired driver and media literacy at a six-week followup. However, no effects were found on decision making, stress management, and rules [58]. This program is relatively easy to implement but is designed ideally to span over five years and thus requires a considerable commitment [14]. 

Behavioral management in the classroom may facilitate prosocial development among older children. The *Good Behavior Game* for 6-7 year olds is a classroom-wide game for contingency management of obtaining a good student role, and reducing disruptive behavior [59]. A rule poster is used (e.g., sitting still, talking in turn, and paying attention), teams are created, and teams are rewarded when all members behave well. Rewards change from tangible and immediate to more abstract and deferred (e.g., from stickers to gold stars). In a randomized design, the use of the Good Behavior Game was found to be associated with improved behavior (less aggressive or disruptive), less alcohol or other drug problems (12% versus 21% control being diagnosed as substance abuse/dependent), and less cigarette smoking, assessed 14 years later, with a stronger impact on males than females [60, 61].

The *Caring School Community Program* (formerly, Child Development Project) is a program designed for elementary school children (kindergarten through sixth grade). This program focuses on children's sense of community towards their school as a means to increase academic motivation and to reduce substance use, violence, and delinquency. This program is purportedly effective when it is able to create an atmosphere at the school that embodies a caring environment. The program consists of three primary components: (1) an intensive classroom component involving cooperative and disciplined learning of reading/language arts; (2) school-wide activities involving teachers, parents, and students in an effort to create a caring school-wide community; and (3) activities for the family that encourage classroom-type activities in the home. Three-year outcomes for a quasi-experimental trial involving closely matched control schools (for baseline third through sixth graders) included relative reduction of current alcohol, cigarette, and marijuana use [62].

The *Know Your Body (KYB) Program* was initially developed in the 1970s [63]. This comprehensive school health promotion program is designed for students in kindergarten through the ninth grade. Educational topics are broad and include exercise, safety, disease prevention, prevention of cigarette smoking, consumer health topics, dental maintenance, HIV/AIDS, substance abuse, and violence prevention. The sessions are organized across five “core skills”: self-esteem, decision making, communication, goal setting, and stress management. Parents are sent letters and community involvement is promoted. Across three randomized controlled trials, significant desired changes in cigarette smoking have been found up to a five-year followup, but no changes have been indicated for marijuana or alcohol use [64].

Improving parenting may help prevent tobacco or other drug misuse among children. *Keep a Clear Mind (KACM)* is a take-home education program designed for older elementary school students (Grades 4 through 6) and their parents [65]. The material in the program consists of weekly sets of activities designed for parents and children to complete together for four weeks. The materials focus on social skills training. This intervention has been shown to positively influence known risk factors for later substance use in a randomized design [66]. This study found that, from pretest to posttest (approximately a four-week period), children in the program condition were more likely than the comparison group to change their expectations of using cigarettes or snuff and to realize that alcohol has harmful effects. In addition, parents from the program condition were more likely than comparison group parents to change their expectations that their child would try alcohol, tobacco, or marijuana. Effects were not found on intention to use drugs. This program is cost effective and easy to implement but requires compliance and time from parents [14].

The *Classroom-Centered (CC) and Family-School Partnership (FSP) Intervention* is a program for first-graders composed of two parts. The CC intervention is designed to target teachers' skills at managing behaviors (e.g., attention problems, aggressive and shy behavior). The FSP component of the intervention targets early risk behaviors by focusing on cultivating communicative relationships about behavior management strategies between parents and teachers [36]. In a randomized control trial with three classrooms: one with the CC intervention, another with the FSP intervention, and the last as a control [67], after six years, it was found that students who were in either the CC or FSP intervention were less likely to begin smoking (26% versus 33%). This program is intensive as it requires participation of school staff and teachers, as well as parents. Training for parents alone takes sixty hours prior to implementation.

*Guiding Good Choices (GGC) *formerly known as* Preparing for the Drug Free Years (PDFY)*, designed for parents of children ages 4 to 11, uses a social development strategy to build bonding, attachment, and commitment by providing opportunities, skills and recognition of individual characteristics [68]. This program was created to prevent teen alcohol, tobacco, and illegal substance use as well as to strengthen parenting skills and family bonding. One randomized controlled study found positive changes in norms against alcohol and other substance use, initiating alcohol use, level of alcohol use, and being drunk up to a 3.5-year followup [69].

The *Seattle Social Development Project (SSDP)* is a prevention program for teachers, parents, and students in Grades 1 to 6 implemented in high crime urban areas [70]. Students and teachers receive mandatory training and parents receive between five and seven optional sessions per year. Teachers are taught to better manage their classrooms; parents are trained in behavior management; students receive social and emotional skills development, and skills for reducing substance use, as well as training to problem solve and learn refusal skills. In a quasi-experimental trial, this program has demonstrated effects in full and later intervention groups relative to a control group on mental and sexual health at a 15-year followup [71]. However, effects on crime and substance use dissipated by then (e.g., effects on alcohol or delinquency initiation had been demonstrated at four-year followup [72]).

The *Linking the Interests of Families and Teachers (LIFT)* is a targeted program that is promising for Grades 1 through 5 [73]. This one hour, twice a week, 10-week program is designed to decrease delinquent behaviors, while promoting positive development in at-risk youth by improving social skills for participants (classroom and playground components) and providing parent training. One randomized controlled study found that compared to control youths, LIFT participants exhibited a reduced average level of use of tobacco, alcohol, and illicit drugs through 12th grade [74]. 

The *Big Brothers Big Sisters of America (BBBSA)* program is designed for at-risk youth ages 6 to 18 [75]. Non-related mentors are matched with children to promote positive development and social responsibility. In the traditional model, the mentor is expected to spend approximately 3 to 5 hours per week with the child for one year. Some of the newer BBBS programs operate slightly differently such that the focus is on establishing school-based mentoring programs where students interact during school hours with their mentor at the school. The program has been shown to be effective on initiation of alcohol and illegal drugs compared to standard care at an 18-month followup of a randomized controlled trial [76].

One unique tobacco use prevention program was of parent-smokers, the *Smoke-free Kids* program for 8-9-year olds [77]. Activity guides, parenting tip sheets, child newsletters, and incentives (e.g., wrist bands and yo-yos) composed the program. Involvement in anti-smoking socialization, support from a health educator, incentives for kids, and communication skills of parents with child at home led to a 12% versus 19% (control condition only received fact sheets) smoking initiation 3-years after baseline in a randomized controlled trial [78].

### 3.3. Young Teens (Approximately 12–15 Years Old)

Tobacco and other drug misuse prevention researchers found intriguing the critical period of young adolescence, in which tobacco and alcohol trial and experimentation increases dramatically [5, 21, 79]. Many researchers have felt that prevention programming should be delivered at this point in child development, during the trial phase of use [21, 80]. Young teens that are at a higher pubertal stage than their peers are relatively likely to experiment with smoking [79], and possibly try other drugs [23]. Young teens who are curious about experiential solutions at the beginnings of their search for identity and who are approached by other teens who share a similar curiosity, may seek out or yield to offers to try drugs or engage in other risky behaviors [21]. Further, personalized risks of smoking tend to decline beginning in the middle schools years, possibly leading to relative overestimates of perceived safety of smoking [81]. Also, prevalence overestimates of tobacco and other drug use exist during this age period [23].

Young teens are ideal candidates for the provision of comprehensive social influences/life skills program material. Drug prevalence overestimates reduction, improvement in difficulties in decision-making, media literacy, and refusal assertion may be strategies that can assist with prevention (initial trial or early use) efforts [82]. Work with family relations still is important although parents are relatively unlikely to serve as a mechanism of friendship selection (they might be trained to do some monitoring). Impact of family programming on young teen cigarette smoking is found in approximately 45% of controlled trials (e.g., see review by Thomas et al. [83], on mostly young teens). Academic remediation may be important. For some young teens, drug abuse cessation treatment will be relevant; treatment could include inpatient stay, intense family therapy, and work on sexual issues as well as drug issues [84]. Most programming has focused on combating social influences, relatively strong antecedents of drug use during young adolescence.

A meta-analysis of 94 controlled trials of school-based substance abuse prevention programs with varying followup (most studies ranged from six-month to two-year followup) suggested that programming for middle school youth might be slightly more likely to achieve a significant effect on alcohol or other drug use than programming for elementary or senior high school youth [85]. Gottfredson and Wilson [85] stated that only for middle school programs “does the evidence clearly imply effectiveness for reducing AOD [alcohol and other drug use]” [85, page 36]. One recent study (randomized in the seventh grade) found that implementation of a social influences drug use prevention program (adapted version of keepin' it REAL) among fifth and seventh grade Mexican heritage youth elicited program effects in eighth grade only among those who received the program in seventh grade [86]; that is, that fifth grade implementation was not important. This study provided support for the suggestion that such programming is more likely to be efficacious among young teens compared to older children.

In summary, key material of instruction that might prevent future tobacco or other drug use among young teens includes: (a) emphasizing counteraction of social influences, (b) provision of life skills (CSI/LS programming), and (c) provision of family skills. Most young teen-directed programs are school-based, which involve social influences/life skills instruction. Specific components of CSI/LS programming include communication skills and refusal assertion, awareness of adult influences, correction of perceived social influence beliefs, activism, decision making, and instruction in physical consequences. Listening and communication skills involve demonstration and modeling of appropriate behavior, behavioral rehearsal, and feedback components. Also, instruction to refuse drug offers (or other behaviors one does not want to do) assertively but not aggressively or passively, is provided as an example of a communication skill. Instructing awareness of large social environmental influences may include media literacy (e.g., understanding misleading social images involved in product depictions). Youth are made aware of advertising influences so that they are less likely to yield to prodrug use images portrayed in the media. Through group activities, like taking group polls on acceptability or prevalence of drug use among peers, youth see drug use is not as widely accepted or prevalent by or among their peers as they perceived. Youth are encouraged to participate in anti-drug use activism (e.g., writing letters to tobacco and alcohol industries) to personalize knowledge, become active learners, and encourage belief change. Decision making and making a public commitment regarding drug use (e.g., not misuse drugs, think about dangers of drug use) also generally are instructed. Finally, often incorporated in comprehensive social influences/life skills programming is brief education on short- and long-term physical consequences of drug use [21, 23].

I describe eight programs developed for young teens. Five of these programs are school based and three are family-based, all on the NREPP list. Six of the programs described are on the older children (6–12 years old) and adolescent (13–17) age group program NREPP search sets. Life Skills Training and Project ALERT were only listed on the adolescent age group program search set (among a total of 120 programs found across both older children and adolescent age groups, only 46 programs appeared in either one website but not the other, indicating high overlap per age group program search set).

*Life Skills Training (LST)* was originally designed for middle/junior high school students [87] and is listed by NREPP for its implementation with young teens [88]. LST includes 15 class sessions in 7th grade, 10 booster sessions in 8th grade, and five booster sessions in 9th grade. Youth are taught personal and social life skills and skills to counteract social influences (e.g., media literacy, managing anxiety, communicating effectively, refusal assertion, and asserting rights). In one randomized controlled trial, follow-up data were collected 6 years later and revealed relatively less cigarette smoking, drunkenness, and combinations of polydrug use (cigarette, alcohol, and marijuana). The strongest effects, across all substances, were obtained among those exposed to at least 60% of the program [88].

More recently, LST has been developed for elementary school students (Grades 3 to 6). This elementary school version also seeks to teach students skills on how to resist peer pressure, as well as competence skills for personal and social situations concerning tobacco and alcohol use [89]. The program consists of 24 classes (30–45 minutes each), over a three-year period (8 classes per year). The program provider is instructed to act more as a coach or skills trainer so as to ensure that personal, interpersonal, and social resistance skills are adequately acquired. In a randomized controlled trial, Botvin and colleagues [89] found that self-esteem was higher, and the annual prevalence rates of smoking and alcohol use were 61% and 25% lower, respectively, at posttest in schools that received the intervention compared to schools that did not receive the intervention three months after the first year of implementation.

The *All Stars* program targets youth from the late elementary school years until high school (ages 11–15 [90]). This program's key outcomes include increased commitment to avoid high-risk behaviors, increased bonding to school and peers, and positive changes in substance use and violence. The mechanisms that the All Stars program uses to promote these changes include creating accurate beliefs about peer norms, altering perceptions on how substance use affects lifestyles, creating commitments to stay substance free and encouraging social and peer bonding. The program is taught by trained teachers in the school classrooms. Training can be completed in person or online and takes eight hours. The 13 core, eight supplemental, and nine booster student activity sessions are each 30–40 minutes long. For the purposes of one evaluation, schools were randomly assigned to two conditions (specialists, standard care), and a teachers condition was added on to make this a partly quasi-experimental trial [91]. A 22-session version of the program was used (14 in class, four with small student assistance groups, and 4 as one-on-sessions). One year post-test results revealed that the program reduced level of cigarette, alcohol, and inhalant use (but not marijuana use) compared to standard care, when delivered by school teachers but not when implemented by specialists (internal delivery agents). Program effects were mediated by two variables: lifestyle incongruence and manifest commitment to avoid risky behavior [91].

*Project ALERT* was designed to be implemented in 11 to 14 year olds [92]. The strategies employed by this program include building school wide norms against substance use, understanding social/health consequences of substance use, identifying prodrug pressure, developing resistance skills, and recognizing the benefits of being drug free. These immediate outcomes are achieved through online teacher training and 14 classroom sessions. Behavioral outcomes achieved in randomized controlled trials include reduced cigarette and marijuana use initiation, decreased current and heavy smoking, and reduced prodrug attitudes and beliefs; a revised version of the program, which added more alcohol prevention material, also demonstrated an impact on alcohol use [93, 94]. The rates of reductions ranged from 19% to 39% [95]. The program tends to work better among baseline non-users, and has revealed effects at 15-month followup [93–95].

*Lions Quest Skills for Adolescence* [96] is a comprehensive life skills education program created for school-wide and classroom instruction in Grades 6 through 8 (10 to 14 year olds), that attempts to instruct social/emotional competence, good citizenship, and positive character. There are 80 45-minute sessions according to the NREPP website, although there are versions with 103 sessions and a condensed version with 40 sessions [97]. One randomized controlled trial that used the 40-session version (three sessions on challenges of entering the teen years, four on building confidence and communication skills, five on managing emotions, eight on peer relationships including resisting peer pressure, and 20 on healthy living and being drug-free), at a one-year followup subsequent to a one-year implementation period, found an impact on marijuana use and binge drinking among baseline binge drinkers [97].

*Project Towards No Tobacco Use* (*Project TNT*) is a tobacco use prevention program that was developed for 7th graders [98]. The theory underlying Project TNT is that youth will best be able to resist using tobacco products if they (1) are aware of misleading social information that facilitates tobacco use (e.g., protobacco advertising, inflated estimates of the prevalence of tobacco use); (2) have skills that counteract the social pressures to achieve approval by using tobacco; and (3) appreciate the physical consequences that tobacco use may have on their own lives. Project TNT is composed of ten core lessons and two booster lessons, 40 to 50 minutes each. Activities include games, videos, role-plays, large and small group discussion, use of student worksheets, homework assignments, activism letter writing, and a videotaping project. The evaluation study contrasted five conditions in a randomized controlled trial, including four curricula and a “usual school health education” control. Three curricula were designed to counteract the effects of separate (single) program components (normative social influence, informational social influence, and physical consequences), whereas a fourth, comprehensive curriculum was designed to counteract all three components. The comprehensive program reduced initiation and weekly use of cigarettes and smokeless tobacco by approximately 30% and 60%, respectively, over the control group, when one-year and two-year follow-up outcomes were averaged together [21, 99, 100].

Prevention materials have also been developed and provided to the family unit among young teens. The focus is on strengthening family dynamics including, for example, instruction in skills training and resource acquisition, family therapy, parent training, contingency management, expressed emotion modification, and social support training. The *Strengthening Families Program: For Parents and Youth 10–14 (SFP 10–14)* has been found to reduce problem behaviors, delinquency, and drug abuse, and to develop social capabilities and school performance in children [101]. The Strengthening Families Program for youth ages 10–14 years of age consists of seven weekly sets of sessions. In 2-hour sessions, parents and youth spend the first hour apart and the second hour together in supervised family activities. Program emphasis is on creating a positive future orientation, age-appropriate expectations and roles (e.g., appropriate disciplinary practices), mutual empathy, and listening to each other. Children also learn peer communication and refusal skills. At a four-year followup of a randomized controlled study among families of 6th graders, Spoth et al. [102] found that 50% of students who received the SFP intervention reported having ever tried alcohol compared to 68% of the control group students; 33% of intervention group students report ever having smoked cigarettes compared with 50% of control group students, and only 7% compared to 17% of control group students reported having ever tried marijuana. In addition, the frequency of alcohol and cigarette use was lower among the intervention group than the control group [102]. SFP also has been effectively implemented with young children [103].

*Family Matters* was developed in the late 1980s [104]. Family Matters is a home-based program designed to prevent tobacco and alcohol use in children 12–14 years old. The program is delivered through four booklets mailed to the home and follow-up telephone calls to parents by health educators. The booklets contain readings and activities designed to get families to consider general family characteristics and tobacco and alcohol use attitudes. Topics discussed include adult supervision and support, rule setting and monitoring, family communication, attachment and time together, education encouragement, family/adult substance use, substance availability, and peer attitudes and media orientation toward substance use. This program was delivered to treatment and control parent-child pairs in a large randomized controlled trial and showed effects on both tobacco and alcohol use at 3 and 12 month followup [105, 106].

*Creating Lasting Family Connections (CLFC*) targets church communities, and high risk 12-to-14 year old teenagers and their parents [107]. In one version of this program, parents of teenagers attend 42 to 56 hours of classes and the teenagers attend 14 to 20 hours of classes, over a four- to seven-month period. Parents' classes are divided into three modules: training on substance abuse knowledge and issues, family management skills, and communication techniques (teenagers joined in on this third module, involving roleplaying). Teens learn about personal and group beliefs pertaining to AOD issues, impact of AOD abuse on the family, and refusal assertion skills. Subsequently a booster (up to six months after the end of training) consisting of bimonthly telephone consultations and/or home visits is implemented. A true experimental evaluation (with an assignment of church-family communities to program or standard care condition) revealed an effect on initiation of alcohol and other drug use (AOD) one year later as moderated by increased AOD knowledge and beliefs consistent with program content, less parent-child conflict, and more parental intolerance of AOD [108].

### 3.4. Older Teens (Approximately 16-17 Years Old)

Dynamic social changes occur between early adolescence (junior high school) and later adolescence (high school). *Older teens* are in the process of solidifying a sense of self and tend to become more resistant to direct influence (e.g., regarding peer influence on smoking [23, 109]). Older teens also tend to socialize in contexts of heterosexual crowds, less mutually dependent on small groups of same-sex peers, and they tend to begin dating and engage in other preparation for an adult lifestyle [110, 111].

Intrapersonal motivations become more important [38]. Yet, older teens exhibit rapid neurobiological changes [110]. In particular, reinforcers may be experienced as relatively rewarding compared to later in adulthood, whereas executive inhibitory processes may not operate as efficiently [23, 110]. For example, the reinforcing value of some behaviors (e.g., kissing, alcohol use) may be much greater than later in adulthood, but there is relatively less inhibitory neocortical functioning in operation. Age-relevant reinforcing behaviors may tend to be associated with each other among older teens. For example, smoking intentions in older adolescence may be related to interest in dating [112]. Intrapersonal motivations tend to dominate as a precursors of risky or health behaviors throughout adulthood (e.g., regarding parental control and refusal self-efficacy [113]). However, older teens may be more sensitive and react negatively to social pressures that contradict their attempts to achieve a sense of self. Thus, instruction in such tobacco and other drug use prevention strategies as refusal assertion training may be received rather negatively by older teens compared to younger or older age groups [38].

While young adolescence has been identified as being the period in which much of smoking initiation occurs [21, 79], older teen non-smokers (e.g., 9th graders) are still susceptible to beginning smoking, particularly if they perceive smoking as resulting in social benefits (e.g., appearing attractive to potential romantic partners) or if they are tolerant of tobacco industry behavior [22]. Tobacco and other drug use may come to serve more as a stress-coping (intrapersonal) function as the substance use acquisition process enters a more advanced phase (regular use). Some researchers suggest that drug abuse prevention programming would be relatively effective if it was implemented when drug use is truly beginning to escalate or become problematic, among older teens for most people [114]. One recent meta-analytic review of 15 studies indicated that prevention of cannabis use was more likely to be demonstrated if incorporating a breadth of prevention programming content (e.g., affect, information, as well as social influences) and if targeting high school youth (14–18 years of age) compared to middle school youth [115]. Possibly, variation of impact of programming by age may vary by drug target. For example, tobacco initiation may be more relevant for prevention efforts among young teens whereas marijuana use initiation may be more relevant for prevention efforts among older teens.

High school-based drug misuse prevention material should be provided so that youth will recall and put into practice relevant key prevention strategies that are relatively likely to involve (a) motivation enhancement, (b) stress-coping skills, and (c) decision making, as opposed to social influences-type material [114]. A motivation/skills/decision-making model is relevant for both prevention and cessation efforts with older teens [23]. Assistance with employment skills and resource acquisition may become quite relevant for older teens. For some older teens, education in parenting skills may be needed.

There are several types of motivation-skills-decision making prevention material that might be utilized. Motivation enhancement material intends to make a youth aware of a discrepancy between his or her behavior and self-perception, leading the youth to desire to bring relatively deviant behavior in line with a generally more favorable self-perception. For example, a youth may view him or herself as a “moderate” type of person, but come to awareness that smoking or other drug misuse is not moderate behavior. Skills instruction includes listening, communication, and self-control skills (particularly in social situations). Decision making skills instruction may help bolster motivation enhancement and life skills instruction [38]. Four programs are presented here. Three are on the NREPP list. Two pertain to drug abuse education within high schools, whereas a third program also involves a parent program and counseling, and the fourth program is computer CD ROM-based and focuses on tobacco use prevention.

*Project Towards No Drug Abuse (TND)* is a drug abuse prevention program for high school youth who are at risk for drug misuse and violence-related behavior [116]. The current version of the Project TND curriculum contains twelve 40-minute interactive sessions taught by teachers or health educators over a 3-week period. Sessions provide instruction in motivation activities to not use drugs; skills in social self-control and coping, communication, and resource acquisition; and decision-making strategies (the “MSD” model [38]). The program also provides one session on tobacco use cessation, with the assumption that most older teens who are smokers are already suffering withdrawal symptoms and are unable to quit. The program has been examined in both traditional and alternative high schools. Prevention effects on 30-day use have been found on hard drug use in all of seven randomized control trials (all of which included a standard care control condition, up to a five-year followup), on alcohol use in four of the trials, and marijuana and cigarette use in only two trials (up to a two-year followup [117, 118]).

The *Reconnecting Youth* (RY) program was implemented among youth at risk for dropout [119, 120]. This program involves 90 sessions within a comprehensive/traditional high school class, delivered generally over a semester, with small student groups and highly trained teachers. Instruction includes use of group support and provision of life skills training (norm setting, self-esteem enhancement, mood management, communication skills, self-monitoring, monitoring goals, school bonding, and social activities), with feedback to parents. Program goals were achieved in one trial through use of a quasi-experimental design, showing relative effects for school performance (18% improvement in grades), drug use (54% decrease in hard drug use), and suicide risk (32% decline in perceived stress).

*Project Schools Using Coordinated Community Efforts to Strengthen Students* (*SUCCESS*) [121] was designed to prevent and reduce substance use among youth 12 to 18 years of age, originally developed for alternative high school youth. The program includes four components: an eight-session drug education program (comprehensive social influences oriented); school-wide activities and materials to increase perceptions of harm and unacceptability of drug use and increase compliance with local policies (e.g., student assistance program); a parent program including education and a parent advisory committee; and brief individual and group counseling and potential referral to appropriate agencies. After one year of implementation of the program in a randomized controlled trial of alternative high schools, impact on alcohol, tobacco and other drug use (ATOD) indicated a 37% relative decrease in prevalence compared to a control group, and 23% of Project SUCCESS youth quit ATOD use compared to only 5% of control comparison schools [122]. A more recent study failed to find a differential impact of use of the program in another experimental design involving alternative high school youth [123].

Finally, *A Smoking Prevention Interactive Experience (ASPIRE)* [124] is a computer-based smoking prevention and cessation curriculum for high school youth [125]. It consists of five weekly sessions in one semester and two booster sessions in the following semester (each 30 minutes long). Subjects either engage in the prevention program if they are nonsmokers or the cessation program if they are current smokers. Module contents are further tailored based on the subject's decisional balance, smoking temptations, depression, and addiction. At an 18-month followup of a randomized controlled trial at inner city high schools, among baseline nonsmokers, smoking initiation rates were lower in the ASPIRE condition than in a standard care control condition which used a self-help booklet (NCI's *Clearing the Air*), 1.9% versus 5.8%. The results for cessation were not significant [125].

### 3.5. Adulthood

Most tobacco and other drug abuse prevention research and programming is implemented among persons 18 years old or younger, partly due to the pragmatics of being able to recruit and follow subjects as well as to the fact that most drug use begins among younger persons [126]. In general, programming for adults is provided to users who have not yet developed a full-blown syndrome of abuse and other negative consequences. A compilation of programs throughout adulthood is available [126, 127].

#### 3.5.1. Emerging Adulthood

The period of *emerging adulthood* (18–25 years old) is one in which youth grapple with the prospects of new opportunities and need to select among them and decide on a firm course in adulthood [128]. During this life transition, individuals achieve relative autonomy from guardians, such as parents, and experience shifts in social roles and normative expectations for their behavior. Emerging adults are typically free from the dependency that characterized childhood (e.g., relatively close parent and teacher guidance), yet are not burdened with the responsibilities of adulthood (e.g., career and parenthood). This freedom allows emerging adults the opportunity to explore diverse potential life directions. Five distinct dimensions of emerging adulthood have been proposed [12, 129, 130]: the age of identity explorations, the age of feeling in-between (not quite an adult), the age of possibilities (optimism), the self-focused age, and the age of instability. The last dimension of emerging adulthood refers to the contradiction that lies with the excitement of exploring life's options, and paradoxically the fear that such a great number of possibilities elicits.

When youth reach 18 years of age in the U.S., young people do take on certain adult roles (e.g., voting) but not others (purchase of alcohol). Young people leaving high school are expected to seek out and select from among new opportunities [13]. These include the following: (1) pursuing and assuming career avenues and financial independence, (2) learning skills of independent living (buying or renting a place to live), (3) growing in self-care skills (cooking, cleaning, grooming, buying goods, traveling), and (4) participating in social adventures (e.g., love and young adult groups). Social adventures may eventually lead to commitment in relationships (e.g., marriage and children).

Youth may also transition from a relatively high level of family conflict in adolescence to the reduction of such conflict in emerging adulthood as they achieve emotional distance from parental demands and regulations and begin to associate more continuously with peers. Successful transitioning through emerging adulthood involves being able to view exploration of life options as positive, hold positive general attitudes about life, become increasingly other-oriented (nurturing), and not feel subjectively caught in-between adolescence and adulthood. For emerging adults, fear or lack of hope that one will be able to satisfactorily settle down into adult roles is a driving source of pressure that might lead one to resort to drug misuse or other self-destructive behavior [13, 130].

Youth that transition poorly through emerging adulthood exhibit unconventional behavior (e.g., drinking alcohol in public and having a child out of a causal affair), unconventional attitudes (e.g., tolerance of deviance), and poor self-control; they may use anger coping and may show interpersonal difficulty. These young people may tend to enter adult roles early (precocious development) prior to being prepared to take on such roles [131]. They tend to drop out of high school or attend part-time education, get married and quickly divorced, become parents while relatively young, and take on undesired full-time employment. They also are relatively likely to exhibit unrestrained drug misuse and abuse.

Key strategies for this developmental stage involve the same motivation-skills-decision-making model used with older teens [38], along with motivational interviewing (MI) strategies, and assistance with helping emerging adults problem-solve means to “settle down” into adulthood [13, 118]. MI is a client-centered counseling style directed at exploring and resolving ambivalence with regard to changing personal behaviors [132]. It differs from other prevention or treatment programming in that its purpose is not to impart information or skills. Rather, it emphasizes exploring and reinforcing participants' intrinsic motivation toward healthy behaviors while supporting autonomy. In a meta-analysis of 39 MI studies of mostly older teens (16–18 years old), Barnett and colleagues [132] found that 67% revealed a positive impact on drug use outcomes at one month to two years followup. In another meta-analysis of 62 studies published between 1985 and 2007 that involved quasi-experimental or experimental designs, interventions for college youth (age range = 18 to 26) that employed MI and personalized feedback showed reductions in drinking and alcohol-related problems (small effect sizes) up to a six-month followup [133].

NREPP lists 34 substance abuse prevention programs for 18–25 year olds; however, only nine of these programs target direct reduction in drug use using controlled research designs (as opposed to another focus such as suicide prevention (e.g., Coping and Support Training or CAST), or training others to deliver drug abuse prevention material as parents (Keep a Clear Mind, Nurse-Family Partnership)). I describe in relative detail two of among the most popular programs used, both on the NREPP website. Both are alcohol abuse prevention programs for college youth, one involving one-on-one counseling and the other involving a computer-based program.

*BASICS (Brief Alcohol Screening and Intervention of College Students)* [134] is aimed at college students 18–24 years old who drink heavily and are at risk for alcohol-related consequences. This program is completed over two structured interviews and is delivered using MI. The first assessment interview gathers information about one's drinking pattern, beliefs, and negative alcohol-related consequences. The second interview, which occurs a week or two later, provides personalized feedback about assessment information (e.g., myths about alcohol's effects, facts on alcohol norms), and ways to reduce future risks associated with alcohol use (behavioral options). BASICS was found to be more effective than an assessment-only normative comparison in reducing alcohol-related problems assessed four years later [135], in a quasi-experimental design. This program is disseminated quite widely in the U.S. [126].

*MyStudentBody.com* targets 18-to-24-year-old college students and is an online, subscription-based program that provides motivational feedback and wellness education about alcohol use and abuse [136]. After logging in, assessment on alcohol use and consequences is conducted followed by immediate tailored feedback based on the assessment. Coping strategies are instructed, and participants are informed about available resources. Also available on this website is an alcohol misuse prevention course for at-risk drinkers. This program is adapted from BASICS. One experimental trial compared the program website to an attention control (access to a website that provided simple education on effects of alcohol) at a three-month followup among persistent heavy drinkers. This study indicated that the program condition delayed increase in average number of drinks per drinking day relative to the control and also indicated a relative reduction in alcohol-related problem behaviors among females only [137].

The seven other prevention programs that were applied to adults on the NREPP list were mostly directed to multiple adult age groups during the working years primarily to reduce heavy or hazardous alcohol use [138]. These included (a) Community Trials Intervention to Reduce High-Risk Drinking (policy-based enforcement including zoning to reduce alcohol outlet density, responsible beverage service, sobriety check points; six-month followup; reductions in drinking and drinking-and-driving in intervention sites relative to control sites), (b) Coping with Work and Family Stress (16 90-minute sessions, involving stress coping, seeking social support, problem solving and communication skills; six-month followup; decreased alcohol use and alcohol use to deal with tension, program versus control), (c) In Shape (one session to motivate a fit social image along with screening, consultation, and tailored feedback; for 18–25 year olds; three-month followup; reductions in alcohol use, drinking-and-driving, and marijuana use [though weak effect], program versus control), (d) Team Awareness (two four-hour sessions among parks and recreation, transportation, and water city employees; coping and help seeking; six-month followup; fewer job-related hangovers, problem drinking and alcohol use frequency, program versus control), (e) Team Resilience (three brief sessions plus one booster; for 18–25 year old restaurant employees; 12-month followup; reduction in recurring heavy drinking, reduction in alcohol-related work problems, program versus control), (f) Training for Intervention ProcedureS (TIPS) for the University (one session; for 18–25 year olds; 11-month followup; reductions in drinking frequency and heavy drinking but effects of program versus control condition dissipated by a 18-month followup), and (g) Wellness Outreach at Work (one screening session that assesses several dimensions of health status including weight, one to four sessions per year for three years; three-year followup; of baseline 9–12 drinks per week drinkers, 38% achieved safe drinking levels versus 22% of controls at the second screening at followup; 65% of baseline smokers were not smoking at that second screening versus 53% of controls).

All of these programs involved controlled trials and achieved impacts (generally short term, such as six months) on alcohol use, though one program also provided an impact on marijuana use (i.e., In Shape) and one program may have provided an effect on cigarette smoking (Wellness Outreach at Work). Most of these programs provided assessment and tailored feedback, and information on dangers of alcohol or other drug use, similar to BASICS. Several also instructed stress-coping skills, help-seeking or help-giving, and two provided fitness (e.g., weight) information (In Shape, Wellness Outreach at Work).

#### 3.5.2. Young-to-Middle Adulthood

Among persons at *young-to-middle adulthood*, between the ages of 26 and 50, there may be many movements/changes in personal and work life (e.g., divorce and remarriage, work promotions). Very few adults initiate legal or illegal drug use after the age of 29 years [126, 139]. Sometimes positive events occur and at other times negative events transpire, any of which may impact on one's drug use. Prevention and cessation strategies that emphasize coping skills and decision making, and provision of legal, medical, and other resources are likely to be relatively important. In addition, group support to help young-to middle-age adults cope with the grief of losing a parent or other older relatives is likely to be important in preventing drug abuse or relapse. Brief interventions such as MI may be relevant to adults in this age range as well as for young adults [126, 140]. Provision of personalized consequences information, random blood or urine testing, approaches that address multiple risk and protective factors (e.g., skills training, education on cardiovascular disease prevention that goes beyond drug misuse), material that is sensitive to the person's daily life, interactive approaches, and longer-term follow through, all may be useful as preventive strategies [141]. Many of the same strategies used for emerging adults are relevant for older adults—though tailoring to developmental tasks specific to the older age range is essential.

“*Healthy Workplace*” is a set of substance abuse prevention interventions for the workplace that are designed for workers who are not substance-dependent, and serve a complementary function to drug testing or employee assistance programs [142]. In some workplace industries (e.g., construction), levels of heavy drinking and illicit substance use are as high as 14% [143] and may average nine percent of the workforce [140]. The five Healthy Workplace interventions—SAY YES! Healthy Choices for Feeling Good, Working People: Decisions About Drinking, the Make the Connection series, Healthy Life 2000 (formerly Prime Life 2000), and Power Tools—target unsafe drinking, illegal drug use, prescription drug use, and healthy lifestyle practices of workers [143, 144]. These programs integrate prevention into broader health promotion programming (stress and nutrition management; in part to be less potentially stigmatizing). Materials are designed to raise awareness of the hazards of substance use and the benefits of healthy behaviors and to teach techniques to live healthier lives. The interventions are delivered in small group sessions using videos and print materials, flexible to different work forces throughout adulthood [145], and they have been found to produce less frequent or lower levels of 30-day drinking and more motivation to drink less compared to standard care up to 11-month followup in randomized trials (e.g., health promotion program alone, or also with substance abuse prevention) or quasi-experimental trials, though, in some trials effects were only found on “stages of change” to drink or smoke (e.g., Power Tools program [143]) and generally were not found on other substances. Other similar programs reveal similar effects, which do tend to be small overall (e.g., Workscreen [140]).

More recently, an interactive website-based alcohol reduction program (*CopingMatters*) was implemented among a heterogenous sample of 145 employees with a three-month followup (1.8% of that workforce, mean age: 40 years old [SD = 11.3]) [146], who had been rated as low or moderate risk for alcohol problems. All subjects received personalized feedback on their levels of stress and coping strategies and were provided with information about alcohol use and its effects. The sample was randomized to receive this material or to a full individualized feedback condition (the sample size in each condition was not found), which involved that material plus feedback on whether they were classified as low, moderate, or high risk for alcohol problems and encouraged to make use of the alcohol use information on the site. An advantage of the full individualized feedback condition was found on decreases in frequency of beer or hard liquor binges at followup.

#### 3.5.3. Older Adulthood

Among the *elderly (older adults)*, approximately 51 years old and older, prescription medication misuse (especially of benzodiazapines, barbiturates, and anti-inflammatory drugs) is the most common form of drug abuse. Many elderly persons frequently ingest two or more prescription medications and may obtain medication from more than one physician. In fact, individuals older than age 65 may average thirteen prescriptions each year [147]. Physicians are not always aware of medications prescribed by others or alcohol use patterns among their patients. The rate of elderly drug misuse and abuse of prescription medications is approximately twice as high as that for other adult age groups, but drug problems may be underestimated or not detected because the elderly are stereotyped as nonusers [147, 148]. Drug abuse estimates among individuals older than 55 years in the United States range from 500,000 to 2.5 million. This relatively low prevalence may reflect a cohort effect (lack of involvement in drug use for a generation), the reduced survivorship of drug abusers, or a maturing-out phenomenon. However, drug abuse in the elderly may increase threefold over the first quarter of the current millennium as those who began use in the 1960s and thereafter continue to remain users into old age [23].

Relatively lower quantities of drug use among the elderly may qualify as drug abuse because of the potential for drugs to exert life-threatening consequences. Drug effects on the elderly may be more long lasting because of relatively slower metabolic rates. Slowing metabolic rates, coupled with ingestion of many medications, may result in an increase in the likelihood of harmful drug interactions. Older adults are subject to many significant life changes that put them at risk for drug dependence. These stressful life changes include retirement, excessive leisure time, and loss of significant others (i.e., loss of social networks and social support [126, 148]). Other life adjustments that place the elderly at risk for drug abuse include potential functional loss and deterioration of physical and mental well-being, often resulting in increased rates of use of prescription medications.

For older adults, one's purview of life as one in which to achieve a subjective sense of wisdom versus a subjective sense of despair [9], as shaped by socioenvironmental experiences, such as amount of free time, lack or imposition of structure, and number of significant others remaining in one's social circles, may drive an individual to resort to constructive or destructive behavior. Programming, that prepares persons for aging (e.g., retirement planning [148]), assists in continuing to help the elderly perceive a sense of social usefulness (e.g., by caretaking for other people or pets, volunteer work, and involvement in the Gray Panthers (activism)), means of enjoyment at a reasonable cost (e.g., which can be obtained through information provided by the American Association for Retired Persons [149] and attendance at elder hostiles), and sense of independence (e.g., good medical care and supportive caretaking from others obtained through reputable agencies) are essential [23, 148]. Also, close medical supervision of medicines being used by the elderly person is essential to prevent unwanted addiction and other consequences. Given the relatively higher frequency of contact, health care providers might be trained to become prevention agents and provide health awareness or aging preparation coping skills [148]. Also, prevention initiatives among the elderly should target improvement in general health behaviors as well as substance misuse or abuse prevention [148], as elderly persons are relatively vulnerable to life threatening conditions relative to other age groups, along with personalized followup.

The evidence-based and clinical literature on formal tobacco or other drug abuse prevention programs for the elderly is practically nonexistent. However, one such program located was an education program, *Aging to Perfection* [126, 150], developed in Florida [151]. This three 90-minute session interactive program targets functional older adults who attend senior centers, community centers and senior residences. The program explores the implications of drug and drug/alcohol-related interactions and safe limits for drinking, instructs problem-solving, and encourages participation in healthy, nonalcohol-related activities and maintaining functional capacity. While disseminated in various locations (e.g., Florida, Oregon, Texas, in the USA), I was unable to locate research support for the program. Other similar programs, which are perhaps less well known and also appear not to be evidence based, include Project MEDS (Medical Education Designed for Seniors; designed to train seniors to give presentations on alcohol and other drugs, and prevention), the Elder-Health Program (drug education for seniors and caregivers), and Senior Sense Speaks (safe medication and alcohol use awareness), as mentioned in Wolfe and Moore [126].

## 4. Summary and Conclusions

For each of the developmental stages presented, there are research and practice supporters and antagonists. That is, there are people who might recommend investment of research resources or implementation time within a particular stage or might argue that resources are better allocated to another stage. Young children are perhaps the most malleable and hence might be expected to be the most important targets for the delivery of tobacco and other drug misuse prevention programming [35]. However, risk for drug use initiation generally is quite distal at that age, and research studies are unable to measure young children long enough to detect effects on drug use behavior. As noted previously, there are reviews suggesting the efficacy or relative efficacy on drug use or misuse of programming delivered among older children [54], young teens [85], or older teens [115].

In each of these developmental periods, program implementers have access to evidence-based programs, or at least credible programming, and may logically consider options for their communities which might include a multiage perspective. I suggest that programming continue at least throughout childhood, adolescence, and emerging adulthood. It also appears that drug abuse problems could occur at any developmental stage throughout the lifespan, and secondary prevention efforts might be important to consider even among the elderly. I do not know that there is a “best” age to deliver tobacco and other drug abuse prevention programming. Numerous effective programs across different age groups were presented in the present review, suggesting the applicability of prevention programming across all ages.

Tobacco or other drug misuse prevention programming is tailored to developmental age periods in two ways. These might be considered tailoring in terms of process and substantive content (see Table 1). Process tailoring pertains to packaging materials in such a way as to address the reading level, cognitive complexity, and learning situations for a developmental stage. Thus, for young children, material may need to be read to them and pictures might be used along with puppets or other such material. Nonabstract analogies might be used to instruct material. Learning situations are likely to occur at home or a preschool/kindergarten environment. For many adults, abstract material involving some reading and possibly homework might be used, involving abstract concepts or descriptions. Learning situations are likely to occur at home or at a worksite or other institutional environment (e.g., church). For the elderly, reading material may or may not be assisted with a larger font.

Content considerations pertain to the “relations” of that developmental stage to tobacco and other drug use. Thus, for young children, little if any mention of harms of drug abuse would be relevant as a prevention strategy. Rather, the focus would be to assist the child to build social connections and emotional control. For many adults, harms of drug abuse instruction would be quite relevant, and other foci might include stress-coping in different life domains and avoiding drug use consequences as opposed to initiation (secondary prevention).

In summary, viewed by developmental stages, young children are likely to receive programming composed of emotional learning techniques, simple information on bad substances (or not), rules or skills to assist with bonding to parents and teachers, and self-control. Instruction emphasizes visual techniques and family counseling as well as education. Older children might be relatively likely to receive drug use physical consequences information, classroom management approaches, as well as additional material to help improve family relations. Young teens are most likely to receive programming that counteracts social influences to use drugs and life skills to pursue prosocial goals. For older teens, and emerging adults, who are focusing on identity development, prevention programming may emphasize motivation- skills-decision making material and possibly includes tobacco use cessation strategies [38]. For adults, prevention strategies tend to emphasize coping skills and decision making, and provision of legal, medical, and other resources. Sometimes counseling to address grief of losing loved ones as a means of prevention, or relapse prevention (a different topic from the present paper), may be helpful [23].

One other, possibly unconsidered perspective that pertains to developmental stage and indirectly to tobacco and other drug abuse prevention is that the addictions may be a problem of lifestyle that is manifested across the lifespan in different types of behaviors [152]. Addiction may refer to cyclical attempts to fulfill appetitive motives, leading to temporary satiation, and eventually to preoccupation, loss of control and negative consequences [153]. The objects of the same “addictive process” may vary across the lifespan. Young children may tend to become addicted to household objects (e.g., blankets, toys, or television), and older children may become addicted to caffeine (e.g., cola sodas) prior to becoming addicted to tobacco or other drugs [154]. Young teens may tend to become addicted to cigarettes, whereas older teens and emerging adults may begin to have difficulties with alcohol and other drugs, and possibly such behaviors as sex, love, the internet, or even exercise [152]. Emerging adults, and young-to-middle-stage adults also may have difficulties across different types of drugs and behaviors, which could extend to workaholism or gambling. Finally, the elderly may tend to grapple with potential addiction to alcohol, prescription medication, and possibly even to medical assistance seeking behavior. A consideration of a wider range of addictive behaviors and the impacts of other deviant behaviors (e.g., use of swear words among young and older children [18]) may help increase our understanding of development, addiction, and how to best prevent tobacco and other drug abuse, and other addictions.

Prevention programming may, in the future, need to consider this wider range of behavior, and focus throughout the lifespan on providing an awareness or prevention of addiction to addictive processes, or to a generic addictive process, that could undermine successful transition through developmental stages. This perspective is not considered in any developmental stage-type prevention research literature, and might be pursued in future research studies. Certainly, consideration of neurobiological (e.g., genetic, brain function) variations, potential variations as a function of gender (e.g., gender roles) or ethnicity (e.g., ethnic identification), or international/cultural variations (e.g., differences in perceptions on when drug use is considered a problem, different age-development based demands in different locations) might also be addressed in future prevention research.

There are several limitations of this review. First of all, examples of studies were provided and no attempt was made to engage in a formal meta-analysis. However, the intent of this paper was to illustrate and provide direction on the process and content of tobacco and other drug use prevention programming throughout the lifespan. Also, a literature search was completed and it seems clear that there is a paucity of work on prevention of tobacco or other drug use among the very young and very old. Second, a solid matrix of developmental tasks by types of prevention programming possibly did not emerge in this review. However, the “backbone” for such a typology was presented (see Table 1). Finally, several potential issues were not discussed. These include the cost-effectiveness of different type of programming for different age groups (which is a serious issue particularly in difficult economic climates), the relative importance of educational programming versus regulatory controls, international translation issues, or whether or not programming should focus on eliciting neurobiological changes in high risk individuals or large social environmental changes. I leave it to future research to expand on these issues. The present paper was the first to my knowledge to lay out tobacco and other drug abuse prevention programming across the lifespan and, hopefully, will stimulate future work in this arena.

## Figures and Tables

**Table 1:** Tobacco and other drug abuse prevention programming as a function of developmental stage.

Developmental stage	Age-appropriate substantive contents	Age-appropriate process examples	Example programs
Young children (0–5 years old)	Attachment enhancement, parenting skills, social and self-control skills, emotional learning, decision making, academic preparation, resource acquisition, maybe simple drug consequences facts	Use of toys, nonverbal demonstration, recognition cards, extensive adult involvement, use of timeout	ICPS, NFP, Fast Track, PATHS, HALO

Older children (6–11 years old)	Social and self-control skills, decision making, emotional learning, academic skills and commitment to school, behavioral management, parenting skills, media awareness, drug consequences	More verbal instruction, attribute-based similarity, rule posters and stickers, involvement with more adults outside the home	BrainTrain4Kids, PY/PM, Good Behavior Game, Caring School Community Program, KYB, KACM, CC/FSP, GGC, SSDP, LIFT, BBBSA, Smoke-free Kids

Young teens (12–15 years old)	Drug prevalence overestimates reduction, normative restructuring, media literacy, refusal assertion, decision making, academic remediation, peer and family communication skills, public commitment, drug consequences	Classroom discussion, classroom peer group interaction, class polls, behavioral rehearsal, role play, homework, games, letter writing	LST, All Stars, Project ALERT, Lions Quest,Project TNT, SFP 10–14, Family Matters, CLFC

Older teens (16–17 years old)	Motivation enhancement, stress-coping skills, self-control and communication skills, decision making and goal monitoring, vocational skills, resource acquisition, drug consequences, tobacco cessation	Talk shows, classroom discussion, peer group interaction, class polls, behavioral rehearsal, games, personal journaling, counseling	Project TND, RY, Project SUCCESS, ASPIRE

Adults: emerging adults (18–25 years old)	Motivational interviewing/enhancement, coping strategies, problem solving, communication skills, drug consequences, “settling-down” material	More personalized and private, assessment, personalized feedback, counseling	BASICS, MyStudentBody

Adults: young-to middle age adults (26–50 years old)	Coping skills, decision making, resource acquisition, grief work, motivational interviewing/enhancement, worksite-related issues	Personalized and private assessment and feedback, material covering more health domains (e.g., diet)	Healthy Workplace, CopingMatters

Adults: older adults (51 years old and older)	Life perspectives, motivation enhancement, resource acquisition, coping skills, problem solving, social usefulness pursuits, medical supervision, multiple health domains, safe limits	Personalized feedback, presentations, group discussion, practice in functional capacity maintenance, use of larger fonts	Aging to Perfection
